# Unraveling the Bidirectional Associations between Parental Knowledge and Children’s Externalizing Behavior

**DOI:** 10.1007/s10964-023-01743-4

**Published:** 2023-02-15

**Authors:** Anke H. Visscher, Savannah Boele, Jaap J. A. Denissen

**Affiliations:** 1 Department of Developmental Psychology, Tilburg University, Tilburg, the Netherlands; 2 Department of Psychology, Education and Child Studies, Erasmus University Rotterdam, Rotterdam, the Netherlands; 3 Department of Developmental Psychology, Utrecht University, Utrecht, the Netherlands

**Keywords:** Externalizing behavior, Parental knowledge, Within-person, Longitudinal study, Random Intercept Cross-Lagged Panel Model

## Abstract

Although within- and between-family bidirectional associations between parental knowledge and children’s externalizing behavior have been theoretically proposed, studies that unravel these associations simultaneously remain scarce. This study examined these bidirectional associations within and between German families. 3611 families participated across one-year intervals between children ages 8 to 15 (50.6% boys, 34.5% fathers, 89.0% German-born, *M*_waves_ = 3.63, *SD*_waves_ = 2.00). Random intercept cross-lagged panel models (RI-CLPM) with linear slopes revealed negative between-family associations between parental knowledge and children’s externalizing behavior, and a negative association between the random linear slopes. Generally, no within-family cross-lagged effects were found, but there were some correlated slopes across families. When teasing apart paternal and maternal knowledge, father-driven but not mother-driven lagged effects of increased knowledge predicting decreased externalizing behavior were found. The findings illustrate the importance of fathers’ knowledge and new directions for within-family studies of parent-child interactions.

## Introduction

Children’s externalizing behavior is often related to maladaptive behaviors and extreme costs for society (Racz & McMahon, 2011). Much research and prevention programs focus on decreasing children’s externalizing behavior. Parental knowledge is widely recognized as a predictor of children’s externalizing behavior (e.g., Lippold et al., 2016; Racz & McMahon, 2011). Parental knowledge can be obtained through parental monitoring, including parental solicitation and behavioral control, which refers to parents’ behaviors aimed at tracking children’s activities (Elam et al., 2017; Liu et al., 2020). Parental knowledge can also be obtained through children’s self-disclosure, which refers to the information about children’s activities that children voluntarily share with their parent(s). Identifying if and how parental knowledge and children’s externalizing behavior are linked may provide valuable insights for prevention and treatment of children’s externalizing behavior. To date, it remains uncertain how parental knowledge and children’s externalizing behavior predict each other over time within families (Boele et al., 2020). Therefore, the current study aimed to investigate whether parental knowledge and children’s externalizing behavior are negatively bidirectionally associated within families over time.

Previous research mainly examined the influence of parenting behavior on children’s externalizing behavior. For example, negative influences of parental knowledge on antisocial behavior one year later were found (Cutrin et al., 2021). Nevertheless, contemporary parenting theories have embraced that children are active agents that influence their parents’ behavior (Pardini, 2008; Soenens et al., 2006). Accordingly, Kerr et al. (2010) proposed that there might be parent-driven processes where parental knowledge affects children’s externalizing behavior, vs. child-driven processes where children’s externalizing behavior affects their parents’ knowledge about their activities and whereabouts. For example, if parents have more knowledge, they are able to monitor and control their children’s behavior more adequately. If children behave more problematic, they often disclose less to their parents which might discourage their parents to ask about their children’s activities leading to a decrease in parental knowledge (Marceau et al., 2015). Thus, increased parental knowledge likely predicts less externalizing behavior, which in turn predicts more parental knowledge. Along these lines, parental knowledge and children’s externalizing behavior most likely influence each other within families.

Research on parental knowledge and children’s externalizing behavior consistently found negative associations across multiple ethnic and socioeconomic backgrounds (e.g., Gryczkowski et al., 2010; Serbin et al., 2015). That is, more parental knowledge was associated with less externalizing behavior and vice versa (e.g., Marceau et al., 2015; Micalizzi et al., 2019). However, these studies mainly investigated the between-family associations in cross-sectional designs, growth curve analyses, or traditional cross-lagged panel models, which fail to assess effects that unfold over time within families (e.g., classic cross-lagged panel models conflate between- and within-family effects; Keijsers & van Roekel, 2018). Effects at the within-family level can reveal whether changes in parental knowledge predict decreases in children’s externalizing behavior within the same family and vice versa. As between-family differences do not necessarily translate to parenting processes that take place within families (Boele et al., 2020; Hamaker et al., 2015), it is vital to also unravel the associations between parental knowledge and children’s externalizing behavior at the within-family level.

Some first studies at the within-family level indeed reveal that related processes can be observed, processes that were consistent with the theory of Kerr and colleagues (2010). For example, one study found support for negative within-family associations between children’s self-disclosure and delinquency, but not between parental solicitation (i.e., parental monitoring) and children’s delinquency (Kapetanovic et al., 2019). This suggested that increases in children’s self-disclosure were followed by subsequent decreases in their delinquent behavior (and vice versa), whereas fluctuations in parental solicitation did not predict fluctuations in their delinquent behavior. Another study found both equally large child-driven and parent-driven effects between children’s externalizing behavior and incompetent parenting (Yan et al., 2021). Additionally, one study found preliminary evidence for bidirectional associations between children’s externalizing behavior and parental monitoring in middle childhood and early adolescence (Elam et al., 2017). Still, these within-family studies did not directly focus on parental knowledge and children’s externalizing behavior.

Moreover, most research focused on brief temporal periods (e.g., three to five measurement waves; Boele et al., 2020) that do not cover longer developmental periods and hence cannot identify long-term stable or changing developmental processes (Liu et al., 2020; Pardini, 2008). Nevertheless, the developmental processes might change during the transition from childhood to adolescence. For example, child-driven effects might increase as children expand their social capabilities and independence, which allows them to enhance their autonomy over their development and preferred environments (Yan et al., 2021). The increase in independence could thus strengthen the child-to-parent influences. In the current study, the developmental period between 8 and 15 years was analyzed. By examining the developmental processes, practical information might be obtained that could inform parent-based interventions, which are often recommended as a cost-effective first-line program for children’s externalizing behavior problems (Weber et al., 2019).

To investigate the dynamic processes between parental knowledge and children’s externalizing behavior, a Random Intercept Cross-Lagged Panel Model (RI-CLPM) with inclusion of random linear slopes was implemented, which shares features with a Latent Curve Model with Structured Residuals (Usami et al., 2019). As an advantage compared to other models, like normal random-intercept cross-lagged panel models or latent change score models, including linear slopes also accounts for the linearly decreasing trend in parental knowledge and children’s externalizing behavior within families (Bongers et al., 2004; Lionetti et al., 2019). This way, it could be examined whether age-specific deviations from the average decreasing linear development of parental knowledge or children’s externalizing behavior were related to later deviations from the average linear development of the other variable. This is important because linear (Bongers et al., 2004; Lionetti et al., 2019) and age-specific development (Keijsers et al., 2010) is likely present in parental knowledge and children’s externalizing behavior when assessing a broad developmental period. For example, one study found that, after controlling for linear development, changes in the level of parental knowledge were related to changes in the level of later children’s externalizing behavior (Lippold et al., 2016).

Next to investigating the bidirectional associations within families, it is important to investigate whether children’s age, children’s gender, and parents’ gender moderate these associations. This could inform parenting programs and future research targeting parental knowledge and children’s externalizing behavior of (non-)susceptible subgroups (Rose et al., 2004). This study explored whether these moderators affect the associations at the between- and within-family levels. First, as partially described above, children’s age might moderate the associations. Parenting behavior likely changes during children’s development: Parental knowledge likely declines as parents acknowledge the increased need for independence and privacy from their children and decrease their efforts to gain knowledge (Lionetti et al., 2019). Additionally, older children might be less likely to accept parental control than younger children, predicting decreases in the parent-to-child influences over time (Koepke & Denissen, 2012). However, few studies investigated age trends over longer developmental periods despite theoretical and practical relevance (Liu et al., 2020). Potentially, developmental differences between late childhood (8–12 years) and early adolescence (13–15 years) might be present (Frick et al., 1999; Lionetti et al., 2019). Such knowledge might ultimately help tailor prevention programs to the needs of different age groups.

Second, children’s gender might moderate the associations (Braza et al., 2015; Yahav, 2007). Girls might be less susceptible to low parental knowledge because they might feel more restricted to display externalizing behavior due to gender-normative expectations, which might cause less necessity for parental regulation of their behavior (Ruiz-Ortiz et al., 2017). Conversely, parental knowledge might have stronger associations with boys’ externalizing behavior as boys might need more parental regulation due to their greater tendency to engage in externalizing behavior (Pinquart, 2017; Willoughby & Hamza, 2011). However, research findings have been inconsistent as some studies found stronger effects for girls (Gryczkowski et al., 2010), some meta-analyses found stronger effects for boys, and other meta-analyses did not find support for moderator effects of children’s gender (Pinquart, 2017). Because these meta-analyses only focused on gender differences in between-family effects, more research on children’s gender differences in within-family effects seemed necessary.

Third, parents’ gender might moderate the associations (McKinney & Renk, 2008; Verhoeven et al., 2010). Mothers’ (on average) greater involvement in children’s upbringing might predict more exposure to children’s behavior, and this exposure might lead to higher levels of mothers’ compared to fathers’ knowledge (Elam et al., 2017). Mothers likely gain more knowledge through increased active monitoring and children’s self-disclosure (Liu et al., 2020). Moreover, Elam and colleagues (2017) found that children’s externalizing behavior only predicted subsequent mothers’ knowledge but not fathers’ knowledge and that mothers’ knowledge predicted subsequent children’s externalizing behavior more strongly than fathers’ knowledge. However, most studies lacked a sufficient sample size to investigate parents’ gender differences accurately, resulting in imprecise knowledge of the moderation (Liu et al., 2020; Zhang et al., 2020).

## Current Study

To investigate the bidirectional associations between parental knowledge and children’s externalizing behavior both within and between families across age, RI-CLPMs with linear slopes were estimated. Consequently, both within-family processes and between-family differences were examined. The current study was pre-registered at https://osf.io/he8sq. Specifically, at the between-family level, it was expected that mean levels of parental knowledge are negatively associated with mean levels of externalizing behavior (Hypothesis 1). Additionally, at the within-family level, it was expected that fluctuations in parental knowledge are negatively associated with subsequent fluctuations in externalizing behavior (Hypothesis 2). Likewise, it was expected that fluctuations in children’s externalizing behavior are negatively associated with subsequent fluctuations in parental knowledge (Hypothesis 3). To visualize all dynamic processes and to prevent model misspecification, all other within- and between-family effects (i.e., the correlations between the slopes and the residuals and the auto-regressive paths), were investigated in an exploratory fashion. Furthermore, children’s age, children’s gender, and parents’ gender were explored as moderators of the associations (Hypothesis 4).

## Methods

### Participants and Procedure

Data from the German *pairfam* study, release 11.0, were used (Brüderl et al., 2020). *Pairfam* is a multi-actor study based on three age cohorts (i.e., 1991–1993, 1981–1983, and 1971–1973), where different family members participate (i.e., main respondent, respondents’ partner, children, and parents). The study started in 2008 with a nationwide random sample from population registers. Approximately 4000 main respondents per cohort were interviewed annually, using a computer-assisted interface. For complete descriptions of the sample and procedures, see Huinink et al. (2011). The ethics committee of the Faculty of Management, Economics, and Social Sciences of the University of Cologne approved the study. Although some studies examined parenting and children’s externalizing behavior using the *pairfam* study (e.g., Hess & Pollmann-Schult, 2020; Zemp et al., 2018), none examined the proposed associations.

From Wave 2 onwards the parenting questionnaire was filled out annually by the main respondents and their partners for every main respondent’s child aged between 8 and 15. Parents’ perspectives on the relationships and interactions with their children and the variables of interest were investigated. Data from Wave 2 till 11 were used, covering the period 2009–2018. Families with data of at least one wave were included. Although reports of children aged 6 and 7 were included from Wave 7 onwards, this study focused on the age range of 8 to 15. Only reports of one parent were used as few families filled out the questionnaire with two parents. This was usually the main respondent: only if reports of the main respondent were missing across all waves, reports of their partners were used (if available).

Eventually, suitable data of at least one wave were available of 3617 children and their parents. After excluding families with no information on children’s age (*N* = 6), data of at least one wave were available of 3611 families (50.6% boys; 34.5% fathers; 89.0% German; 2.9% Turkish; 1.7% Russian; 1.5% Polish, 4.9% Other). Each wave contained data of 689 to 1750 families. When structured according to age, each year contained data of 848–1836 families. Families participated on average 3.63 times (*SD* = 2.00). According to the *N*:q rule (Kline, 2015), the recommended ratio between the cases and free parameters is at least 20:1. Following this rule, inclusion of minimum 1480 families for the RI-CLPM and 2960 families for the multigroup RI-CLPM was necessary, as maximal 74 and 148 free parameters in the RI-CLPM and multigroup RI-CLPM were estimated. As data were available of 3611 families, the sample size was deemed appropriate.

### Measures

#### Parental knowledge

Parental knowledge was measured using the subscale Monitoring, which includes items based on the subscale Poor Monitoring/Supervision of the Alabama Parenting Questionnaire (Essau et al., 2006; Reichle & Franiek, 2009). The subscale contained four items, indicating the degree to which parents were informed about their children’s activities and social contacts. Parents answered on a 5-point Likert scale ranging from 1 (*never*) to 5 (*very often*). Based on item semantics, two subscales were further explored. Two items seemed to indicate parental solicitation (i.e., “You discuss with your child about his/her new friends”, “You ask your child when he/she went out”) and two items seemed more direct indicators of parental knowledge (i.e., “You know exactly where your child is when he/she goes out”, “You get to know them soon when your child makes new friends”). A confirmatory factor analysis with data of wave 2 showed a lack of fit of this possible two-factor structure (comparative fit index [CFI] = 0.95, Tucker-Lewis index [TLI] = 0.72, root-mean-square error of approximation [RMSEA] = 0.20, standardized root-mean-square residual [SRMR] = 0.04). Therefore, likewise to Lux and Walper (2019), analyses were conducted with the original scale. Scale scores were calculated by averaging the item scores. Higher scores indicated higher levels of parental knowledge, respectively.

Internal consistency at the between-family level was overall acceptable, with alpha coefficients ranging from 0.72 to 0.78 across waves. The average inter-item correlation across waves was 0.43 (*r*s = 0.39–0.47), indicating representative items of the same construct while capturing a slightly smaller bandwidth of parental knowledge (Piedmont, 2014). Analyses indicated that the intraclass correlation coefficient (ICC) of the intercept of parental knowledge was 0.59. In other words, 59% of the total variance across ages 8 to 15 could be contributed to stable between-family differences and 41% to annual within-family fluctuations.

#### Children’s externalizing behavior

Children’s externalizing behavior was measured using the subscale Conduct Problems from the Strengths and Difficulties Questionnaire (Klasen et al., 2003). The subscale contained five items, indicating parents’ perspectives of children’s externalizing behavior. Parents answered items like “Often loses temper” and “Steals from home, school or elsewhere” on a 3-point Likert scale ranging from 1 (*not true*) to 3 (*certainly true*). After recoding the second item, scale scores were calculated by averaging the item scores. Higher scores indicated higher levels of externalizing behavior, respectively.

Internal consistency at the between-family level was overall moderate, with alpha coefficients ranging from 0.55 to 0.63 across waves. The average inter-item correlation across waves was 0.23 (*r*s = 0.21–0.27), indicating representative items of the same construct (Piedmont, 2014). The ICC of the intercept was 0.58. In other words, 58% of the total variance across ages 8 to 15 could be contributed to stable between-family differences and 42% to annual within-family fluctuations.

### Data Analysis

Preliminary analyses including descriptive statistics and bivariate correlations were performed in R, Version 4.0.3. First, the assumptions of normality and missing data were tested (Kumar, 2015). Patterns in missingness were tested with Little’s test in SPSS, Version 26.0, and visualized in R. As data were missing completely at random at every wave (χ^2^/*df* < 3, 0.00–5.58%), no additional analyses were performed. Additionally, no variables were transformed as the skewness coefficients ranged between -0.95 and 1.33, and visual inspections showed no heavily skewed distributions. Maximum likelihood (ML) estimator was used in the main analyses as no skewed distributions were present and the Likert-scaled subscales could be treated as interval scales (Norman, 2010). Measurement invariance tests showed that, when boys were compared to girls on the second wave, residual invariance was supported for both parental knowledge and children’s externalizing behavior (*p* > 0.05). All following analyses were performed with Lavaan, version 0.6-7, in R (Rosseel, 2012).

**Fig. 1:**
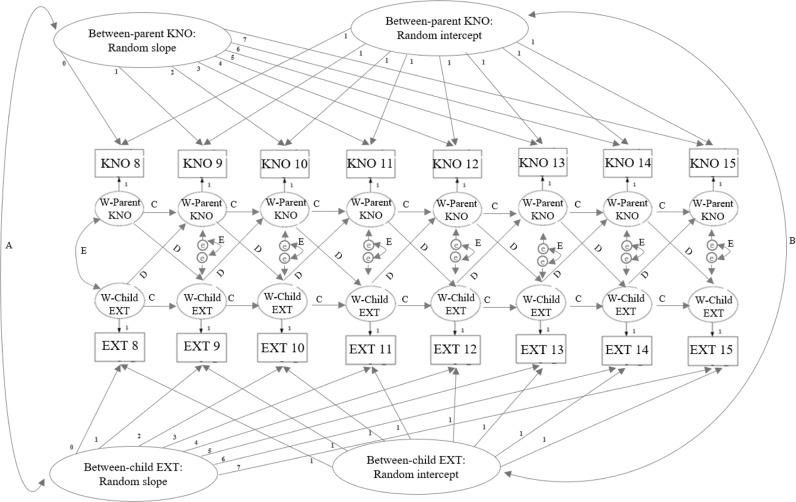
Proposed RI-CLPM with linear slopes. *Note*. A = Intercept-intercept correlation. B = Slope-slope correlation. C = Autoregressive paths. D = Cross-lagged paths. E = Correlated residuals

Data were sorted by children’s age instead of wave to examine the bidirectional associations more accurately across children’s age, as families could start participating during different waves when children turned 8 years. Bivariate correlations were examined at the between- and within-family level. As the ICCs for parental knowledge and children’s externalizing behavior showed more than 10% within-family variance, a RI-CLPM was deemed appropriate. First, the RI-CLPM was specified with two intercepts, with loadings (set to one) onto the observed measurements of parental knowledge and children’s externalizing behavior at each age. Intercepts were included to account for constant age-invariant individual differences and reflected between-family differences in mean levels of the variables across age (Hypothesis 1). Two slopes were included, with loadings (set to 0, 1, 2, 3, 4, 5, 6, and 7) onto the observed measurements of parental knowledge and children’s externalizing behavior to account for linear age trends in these variables. Correlated intercepts were specified to control for between-family covariation. Correlated slopes were specified to control for between-family linear co-development between parental knowledge and children’s externalizing behavior. The observed measurements were regressed onto their latent factors. These factor loadings were set to one. Additionally, autoregressive and cross-lagged paths were specified between these latent factors. The cross-lagged paths indicated the within-family effects of age-specific fluctuations in parental knowledge and children’s externalizing behavior on subsequent fluctuations in the other variable (Hypothesis 2, Hypothesis 3). The autoregressive paths indicated within-family carry-over effects (i.e., when previous fluctuations in the family-specific mean levels predicted subsequent fluctuations in the mean levels). Additionally, age-invariant (residual) variances at the within-family part were included and correlated and indicated age-specific fluctuations in the family-specific means. The correlated residuals indicated the correlation between the residual variance at each age. See Fig. 1 for an overview of the RI-CLPM. Model fit was considered acceptable if the CFI and TLI were above 0.90, and the RMSEA and SRMR were below 0.08 (Byrne, 2012).

Subsequently, multiple models were estimated with constrained parameters across age in the following order: constrained correlated residuals, autoregressive paths, and cross-lagged paths. These models were compared to retain the most parsimonious model with the RI-CLPM without constraints as baseline model. In the first step, the model without constraints was compared to a model with constrained correlated residuals. χ^2^-difference tests were conducted. If the test was significant (*p* < 0.05), age effects were assumed to be present and the model without (extra) constraints was used for further model comparisons. If not significant (*p* > 0.05), no age effects were assumed to be present, and the age-constrained model was used in further model comparisons. See Table 2 for the final order of the model comparisons.

Additionally, multi-group RI-CLPMs were examined to explore parents’ and children’s gender as moderators (Hypothesis 4). First, multi-group RI-CLPMs of the final RI-CLPM were run as baseline models. Thereafter, as the model is sensitive for model misspecification, the multi-group RI-CLPM was compared to identical multi-group RI-CLPMs with parameters constrained across gender in the following order: constrained intercept-intercept correlation, slope-slope correlation, correlated residuals, auto-regressive paths, and cross-lagged paths. χ^2^-difference tests were used to investigate gender differences with the multi-group RI-CLPM without constraints as the baseline model. If the comparison test was significant, gender differences were assumed to be present in the specific parameter and the model without (extra) constraints was used for further model comparisons. If not significant, no gender effects were assumed to be present and the model with constraints was used in further model comparisons.

## Results

### Preliminary Analyses

Table 1 shows the descriptives of parental knowledge and children’s externalizing behavior per age and gender (i.e., *n*, *M, SD*). For an overview of the descriptive statistics per wave, see Supplementary Table 1. Between-family correlations between parental knowledge, children’s externalizing behavior, parents’ gender, and children’s gender are displayed in Supplementary Table 2. Overall, consecutive measurements of externalizing behavior (*r* between 0.33 and 0.66), and parental knowledge (*r* between 0.42 and 0.66) were moderately stable across age. Annual measurements of parental knowledge and externalizing behavior were, on average, weakly correlated (*r* between −0.25 and −0.02).

**Table 1:** Descriptive statistics per participants’ age for the total sample and gender

	Sample size			Descriptive statistics									
				Total		Boys		Girls		Fathers		Mothers	
	*n*	*n*_boys_	*n*_fathers_	*M*	*SD*	*M*	*SD*	*M*	*SD*	*M*	*SD*	*M*	*SD*
Parental knowledge													
Age 8	1742	882	604	4.23	0.56	4.20	0.56	4.26	0.55	3.95	0.57	4.38	0.49
Age 9	1823	931	612	4.20	0.57	4.14	0.60	4.26	0.53	3.94	0.59	4.33	0.51
Age 10	1783	907	604	4.15	0.57	4.10	0.57	4.20	0.56	3.91	0.59	4.27	0.51
Age 11	1657	851	536	4.08	0.58	4.05	0.57	4.11	0.58	3.85	0.62	4.19	0.52
Age 12	1455	751	462	4.04	0.59	3.99	0.60	4.10	0.57	3.78	0.64	4.16	0.53
Age 13	1270	649	393	3.95	0.60	3.91	0.61	4.00	0.59	3.75	0.63	4.04	0.57
Age 14	1074	543	334	3.86	0.62	3.81	0.60	3.91	0.63	3.62	0.63	3.97	0.58
Age 15	848	434	242	3.79	0.65	3.73	0.66	3.86	0.63	3.58	0.64	3.87	0.63
Externalizing behavior													
Age 8	1753	883	607	0.36	0.32	0.40	0.32	0.31	0.30	0.38	0.32	0.34	0.32
Age 9	1836	938	616	0.35	0.31	0.40	0.33	0.31	0.29	0.37	0.32	0.34	0.31
Age 10	1791	909	605	0.35	0.32	0.39	0.33	0.31	0.29	0.36	0.32	0.35	0.31
Age 11	1663	852	536	0.35	0.32	0.39	0.32	0.31	0.31	0.35	0.31	0.35	0.32
Age 12	1460	755	462	0.36	0.32	0.40	0.34	0.32	0.30	0.37	0.32	0.36	0.32
Age 13	1271	649	393	0.35	0.32	0.37	0.32	0.33	0.31	0.31	0.30	0.36	0.32
Age 14	1077	545	335	0.33	0.31	0.35	0.31	0.32	0.30	0.34	0.31	0.33	0.31
Age 15	850	434	242	0.33	0.31	0.33	0.31	0.33	0.31	0.34	0.30	0.32	0.32

Pooled *SD* for parental knowledge for parents’ gender = 0.58. Pooled *SD* for parental knowledge for children’s gender = 0.60. Pooled *SD* for children’s externalizing behavior for parents’ gender = 0.32. Pooled *SD* for children’s externalizing behavior for children’s gender = 0.31

Linear mixed-effects models (lmerTest package) showed that the expected average value (intercept) was 4.53 for parental knowledge (*p* < 0.001). Observations of parental knowledge decreased by 0.07 units per age increment (*p* < 0.001). Although no interaction between children’s and parents’ gender was found (*p* = 0.214), parents of boys had (on average) less knowledge than parents of girls (*b* = 0.05, *p* = 0.046), and fathers less knowledge than mothers (*b* = 0.37, *p* < 0.001). For children’s externalizing behavior, the expected average value (intercept) was 0.45 (*p* < 0.001), and observations decreased by 0.01 units per age increment (*p* < 0.001). There were differences between boys and girls, where girls had (on average) lower levels of externalizing behavior than boys (*b* = −0.07, *p* < 0.001). No differences in reports of mothers and fathers (*p* = 0.378) and no interactions between children’s and parents’ genders (*p* = 0.139) were found.

### Model Selection

**Fig. 2:**
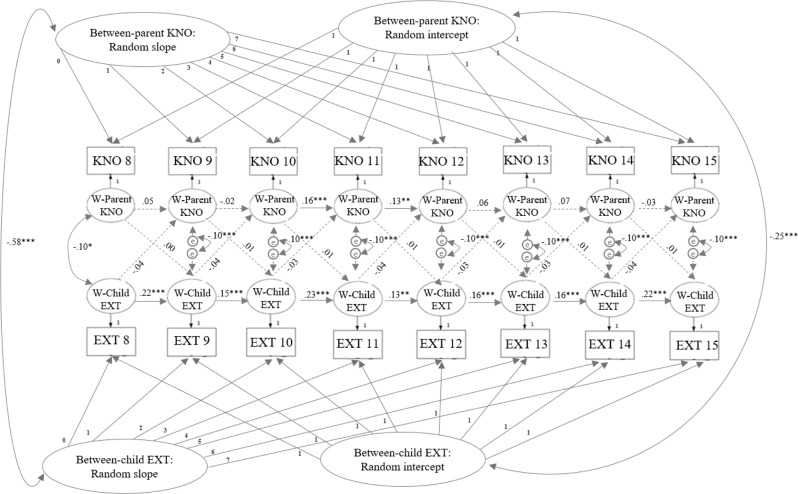
Model results of the RI-CLPM with constrained correlated residuals and cross-lagged paths across age. *Note*. Statistics are standardized parameter estimations. KNO Parental knowledge, EXT Children’s externalizing behavior, W Within. Dotted lines represent nonsignificant relations. Bold lines represent significant relations. *p < 0.05. **p < 0.01. ***p < 0.001

Since the fit measures of the RI-CLPM (Model 1) indicated good fit to the data, the most parsimonious model was retained, see Table 2. If the model with extra time constraints did not fit the data significantly worse, no age differences were found, and further analyses were conducted with the model with extra time constraints. Eventually, parameter estimates of the RI-CLPM with constrained correlated residuals, constrained cross-lagged paths, and unconstrained stability paths were investigated (Model 4), see Fig. 2 and Table 3. For an overview of the intercept and slope variances, and their associations of the performed models, see Table 4.

**Table 2:** Model summary statistics and model comparisons of the RI-CLPMs

Model	χ^2^	*df*	CFI	TLI	RMSEA	SRMR	Model comparison	Δχ^2^	Δ*df*	*p*
1. RI-CLPM	191.35	78	0.99	0.98	0.02	0.05				
2. Con. correlated residuals	208.71	96	0.99	0.99	0.02	0.05	1 vs. 2	17.36	18	0.498
3. Con. autoregressive paths	234.42	108	0.99	0.99	0.02	0.05	2 vs. 3	25.71	12	0.012
4. Con. cross-lagged paths	222.95	108	0.99	0.99	0.02	0.05	2 vs. 4	14.24	12	0.286
5. MG children’s gender	377.94	216	0.98	0.98	0.02	0.06				
6. Con. intercept-intercept correlation	378.29	217	0.98	0.98	0.02	0.06	5 vs. 6	0.36	1	0.550
7. Con. slope-slope correlation	378.35	218	0.98	0.98	0.02	0.06	6 vs. 7	0.06	1	0.805
8. Con. correlated residuals	388.29	221	0.98	0.98	0.02	0.06	7 vs. 8	9.94	3	0.019
9. Con. autoregressive paths	479.75	233	0.97	0.97	0.02	0.08	7 vs. 9	101.40	15	<0.001
10. Con. cross-lagged paths	379.21	220	0.98	0.98	0.02	0.06	7 vs. 10	0.86	2	0.651
11. MG parents’ gender	349.49	216	0.99	0.98	0.02	0.06				
12. Con. intercept-intercept correlation	349.75	217	0.99	0.98	0.02	0.06	11 vs. 12	0.26	1	0.610
13. Con. slope-slope correlation	349.92	218	0.99	0.98	0.02	0.06	12 vs. 13	0.16	1	0.687
14. Con. correlated residuals	381.34	221	0.98	0.98	0.02	0.06	13 vs. 14	31.43	3	<0.001
15. Con. autoregressive paths	481.84	233	0.97	0.97	0.02	0.08	13 vs. 15	131.92	15	<0.001
16. Con. cross-lagged paths	358.81	220	0.99	0.98	0.02	0.06	13 vs. 16	8.90	2	0.012

All χ^2^-statistics were significant, *p* < 0.001

*RI-CLPM* Random-Intercept Cross-Lagged Panel Model, *Con.* Constrained, *MG* Multigroup, *CFI* Comparative fit index, *TLI* Tucker-Lewis index, *RMSEA* Root-mean-square error of approximation, *SRMR* standardized root-mean-square residual, CFI, TLI, RMSEA, and SRMR are model fit measures

**Table 3:** Parameter estimates of the RI-CLPM with constrained correlated residuals and cross-lagged paths across age

Parameter	*B*	*SE B*	β	*z*	*p*	95% CI for *B*
Autoregressive paths						
Ext. Age 8 → Ext. Age 9	0.22	0.04	0.22	5.48	<0.001	[0.14, 0.30]
Ext. Age 9 → Ext. Age 10	0.15	0.04	0.15	4.19	<0.001	[0.08, 0.22]
Ext. Age 10 → Ext. Age 11	0.24	0.04	0.23	6.40	<0.001	[0.17, 0.31]
Ext. Age 11 → Ext. Age 12	0.13	0.04	0.13	3.32	0.001	[0.05, 0.20]
Ext. Age 12 → Ext. Age 13	0.16	0.04	0.16	3.52	<0.001	[0.07, 0.25]
Ext. Age 13 → Ext. Age 14	0.16	0.05	0.16	3.52	<0.001	[0.07, 0.26]
Ext. Age 14 → Ext. Age 15	0.22	0.06	0.22	3.92	<0.001	[0.11, 0.33]
Kno. Age 8 → Kno. Age 9	0.05	0.05	0.05	1.00	0.317	[−0.05, 0.15]
Kno. Age 9 → Kno. Age 10	−0.02	0.04	−0.02	−0.47	0.636	[−0.09, 0.06]
Kno. Age 10 → Kno. Age 11	0.16	0.04	0.16	4.32	<0.001	[0.09, 0.24]
Kno. Age 11 → Kno. Age 12	0.13	0.04	0.13	3.45	0.001	[0.06, 0.20]
Kno. Age 12 → Kno. Age 13	0.06	0.04	0.06	1.47	0.143	[−0.02, 0.15]
Kno. Age 13 → Kno. Age 14	0.07	0.05	0.07	1.51	0.132	[−0.02, 0.16]
Kno. Age 14 → Kno. Age 15	−0.03	0.05	−0.03	−0.46	0.647	[−0.13, 0.08]
Cross-lagged paths						
Ext. Age 8 → Kno. Age 9	−0.06^a^	0.03	−0.04	−1.95	0.051	[−0.12, 0.00]
Ext. Age 9 → Kno. Age 10	−0.06^a^	0.03	−0.04	−1.95	0.051	[−0.12, 0.00]
Ext. Age 10 → Kno. Age 11	−0.06^a^	0.03	−0.03	−1.95	0.051	[−0.12, 0.00]
Ext. Age 11 → Kno. Age 12	−0.06^a^	0.03	−0.04	−1.95	0.051	[−0.12, 0.00]
Ext. Age 12 → Kno. Age 13	−0.06^a^	0.03	−0.03	−1.95	0.051	[−0.12, 0.00]
Ext. Age 13 → Kno. Age 14	−0.06^a^	0.03	−0.03	−1.95	0.051	[−0.12, 0.00]
Ext. Age 14 → Kno. Age 15	−0.06^a^	0.03	−0.04	−1.95	0.051	[−0.12, 0.00]
Kno. Age 8 → Ext. Age 9	0.00^b^	0.01	0.00	0.30	0.765	[−0.02, 0.02]
Kno. Age 9 → Ext. Age 10	0.00^b^	0.01	0.01	0.30	0.765	[−0.02, 0.02]
Kno. Age 10 → Ext. Age 11	0.00^b^	0.01	0.01	0.30	0.765	[−0.02, 0.02]
Kno. Age 11 → Ext. Age 12	0.00^b^	0.01	0.01	0.30	0.765	[−0.02, 0.02]
Kno. Age 12 → Ext. Age 13	0.00^b^	0.01	0.01	0.30	0.765	[−0.02, 0.02]
Kno. Age 13 → Ext. Age 14	0.00^b^	0.01	0.01	0.30	0.765	[−0.02, 0.02]
Kno. Age 14 → Ext. Age 15	0.00^b^	0.01	0.01	0.30	0.765	[−0.02, 0.02]
Correlations						
Correlation Age 8	−0.01	0.00	−0.10	−2.53	0.011	[−0.01, −0.00]
Residual correlation Age 9	−0.01^c^	0.00	−0.10	−6.95	<0.001	[−0.01, −0.01]
Residual correlation Age 10	−0.01^c^	0.00	−0.10	−6.95	<0.001	[−0.01, −0.01]
Residual correlation Age 11	−0.01^c^	0.00	−0.10	−6.95	<0.001	[−0.01, −0.01]
Residual correlation Age 12	−0.01^c^	0.00	−0.10	−6.95	<0.001	[−0.01, −0.01]
Residual correlation Age 13	−0.01^c^	0.00	−0.10	−6.95	<0.001	[−0.01, −0.01]
Residual correlation Age 14	−0.01^c^	0.00	−0.10	−6.95	<0.001	[−0.01, −0.01]
Residual correlation Age 15	−0.01^c^	0.00	−0.10	−6.95	<0.001	[−0.01, −0.01]
Intercept-intercept correlation	−0.03	0.00	−0.25	−9.41	<0.001	[−0.03, −0.02]
Slope-Slope correlation	−0.00	0.00	−0.58	−4.90	<0.001	[−0.00, 0.00]

Superscripts indicate that parameters could be constrained as equal across age

*Kno.* Parental knowledge, *Ext.* Children’s externalizing behavior, *CI* Confidence interval

**Table 4:** Intercept variances, slope variances, and their associations of the RI-CLPMs

Model	*s*^2^	*SE*	*p*	s^2^	*SE*	*p*	*r*	*cov*.	*SE*	*p*
	Intercept parental knowledge			Intercept externalizing behavior			Associations between intercepts			
1. RI-CLPM	0.19	0.01	<0.001	0.05	<0.01	<0.001	−0.25	−0.03	<0.01	<0.001
2. RI-CLPM: Con. correlated residuals	0.19	0.01	<0.001	0.05	<0.01	<0.001	−0.25	−0.03	<0.01	<0.001
3. RI-CLPM: Con. autoregressive paths	0.19	0.01	<0.001	0.05	<0.01	<0.001	−0.25	−0.03	<0.01	<0.001
4. RI-CLPM: Con. cross-lagged paths	0.19	0.01	<0.001	0.05	<0.01	<0.001	−0.25	−0.03	<0.01	<0.001
	Slope parental knowledge			Slope externalizing behavior			Associations between slopes			
1. RI-CLPM	<0.01	<0.01	<0.001	<0.01	<0.01	<0.001	−0.53	<0.01	<0.01	<0.001
2. RI-CLPM: Con. correlated residuals	<0.01	<0.01	<0.001	<0.01	<0.01	<0.001	−0.56	<0.01	<0.01	<0.001
3. RI-CLPM: Con. autoregressive paths	<0.01	<0.01	<0.001	<0.01	<0.01	<0.001	−0.56	<0.01	<0.01	<0.001
4. RI-CLPM: Con. cross-lagged paths	<0.01	<0.01	<0.001	<0.01	<0.01	<0.001	−0.58	<0.01	<0.01	<0.001

*RI-CLPM* Random intercept cross-lagged panel model, *Con* Constrained, *cov.* Covariance

### Between-Family Results

To examine whether mean levels of parental knowledge and children’s externalizing behavior were negatively associated (Hypothesis 1), the intercept-intercept correlation of Model 4 was examined. A moderate negative correlation indicated that these mean levels were negatively correlated (β = −0.25, *p* < 0.001), which supports the first hypothesis. The linear trends showed a negative slope-slope correlation, indicating a negative association between linear changes (β = −0.58, *p* < 0.001). In other words, families who had a steeper increase in parental knowledge also had a steeper decrease in children’s externalizing behavior during the study period than families with a less steep increase in parental knowledge.

### Within-Family Results

To examine whether age-specific fluctuations in the family-specific linear development of parental knowledge and children’s externalizing behavior were negatively associated with subsequent fluctuations in the family-specific linear development in the other variable (Hypothesis 2, Hypothesis 3), the cross-lagged paths of Model 4 were investigated. The cross-lagged paths, which were constrained to be equal across age, were not significant (*p* > 0.05). This indicated an absence of (statistically significant) within-family reciprocal effects of age-specific fluctuations in the family-specific linear development in parental knowledge and externalizing behavior. Likewise, no differences in these paths were found between ages 8–12 and 13–15.

Furthermore, the within-family effects were controlled for correlated residuals and carry-over effects and were investigated exploratory. The residuals showed a significantly negative correlation that could be constrained across age (β = −0.10, *p* < 0.001). The autoregressive paths of children’s externalizing behavior were all significantly positive, although modest in size (Model 4, βs = 0.13–0.23), indicating within-family carry-over effects across the one-year intervals between ages 8 to 15. For parental knowledge, the autoregressive paths between ages 10–11 (β = 0.16, *p* < 0.001), and 11–12 (β = 0.13, *p* = 0.001) were significant, positive, and modest in size, indicating carry-over effects between ages 10 to 12.

### Moderating Role of Children’s and Parents’ Gender

**Fig. 3:**
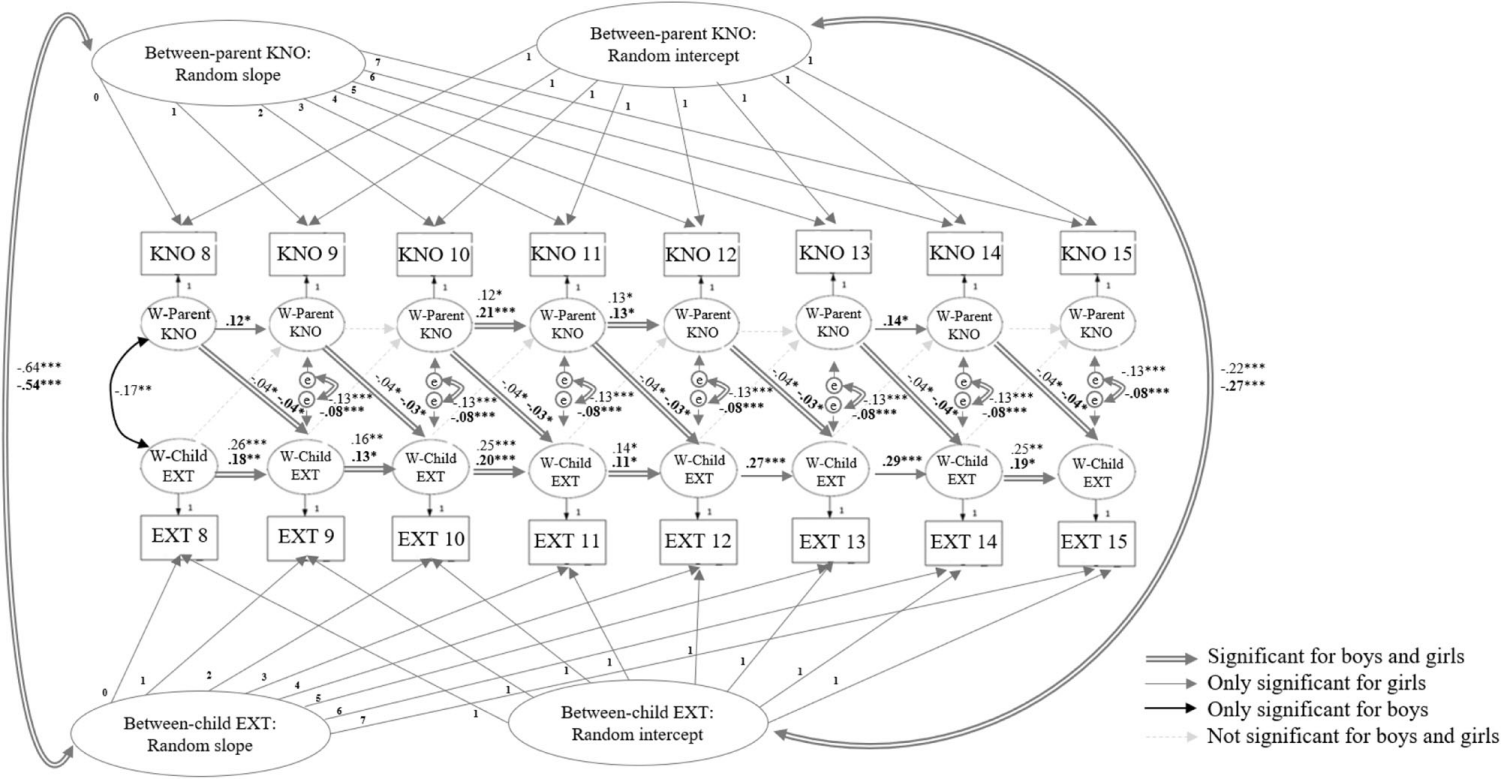
Model results of the final multi-group RI-CLPM for children’s gender. *Note*. Statistics are significant standardized parameter estimations. KNO Parental knowledge, EXT Children’s externalizing behavior, W Within. Estimates in bold are girls’ estimates, estimates in roman are boys’ estimates. *p < 0.05; **p < 0.01; ***p < 0.001

**Fig. 4:**
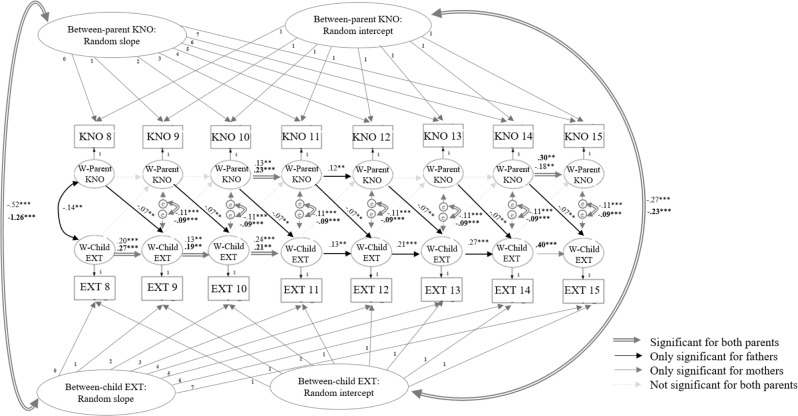
Model results of the final multi-group RI-CLPM for parents’ gender. *Note*. Statistics are significant standardized parameter estimations. KNO Parental knowledge, EXT Children’s externalizing behavior, W Within. Estimates in bold are mothers’ estimates, estimates in roman are fathers’ estimates. *p < 0.05; **p < 0.01; ***p < 0.001

As this study aimed to explore moderation at the intercept-intercept correlation and cross-lagged effects only, the description of model selection and moderation effects at other effects (i.e., the slope-slope correlation, correlated residuals, auto-regressive paths) are described in Supplementary Text 1 and Supplementary Tables 3–6. For children’s gender, no gender differences in the negative association between mean levels of parental knowledge and children’s externalizing behavior were found, as the intercept-intercept correlation could be constrained across gender, see Table 2 and Fig. 3. Likewise, no children’s gender differences were found for the constrained cross-lagged paths^1^. For parental gender, no gender differences were found in the negative association between the mean levels as the intercept-intercept correlation could be constrained across gender, see Table 2 and Fig. 4. Similarly, no parental gender differences were found in terms of the (insignificant) cross-lagged association between externalizing behavior and subsequent parental knowledge. Nevertheless, parental gender differences were found for the cross-lagged paths, as age-specific fluctuations in parent-specific mean levels of parental knowledge were negatively associated with subsequent age-specific fluctuations in child-specific mean levels of externalizing behavior (Hypothesis 2) for fathers (β = −0.07, *p* = 0.001), but not for mothers.

### Sensitivity Analyses

As sensitivity analyses, additional (not pre-registered and thus exploratory) RI-CLPMs with incorporation of the correlations between the intercepts and slopes were run. Multiple models were estimated with constrained parameters in the identical order as described above for the main models. See Supplementary Text 2 and Supplementary Tables 7 and 8, for model results and their descriptions. Model comparisons and model estimates showed seemingly similar results compared to the main models.

## Discussion

Bidirectional processes between parental knowledge and children’s externalizing behavior across children’s development are proposed by some theories (e.g., Kerr et al., 2010). According to these perspectives, decreased parental knowledge may contribute to later increased children’s externalizing behavior and vice versa. As these processes occur over time within individual families, these bidirectional lagged associations were investigated in random intercept cross-lagged panel models (RI-CLPM) that included linear slopes. Both within-family processes and between-family differences were examined. Children’s age, gender, and parents’ gender were explored as moderators of the within-family lagged associations.

The results of the current study demonstrated negative between-family correlations, indicating that children in families with higher mean levels of parental knowledge had lower mean levels of children’s externalizing behavior than children in families with lower mean levels of parental knowledge (Hypothesis 1 confirmed). Nonetheless, at least for the combined sample of mothers and fathers, age-specific fluctuations in parent-specific mean levels of parental knowledge did not predict subsequent age-specific fluctuations in child-specific mean levels of externalizing behavior within families (Hypothesis 2 not confirmed). In the reverse direction, fluctuations in children’s externalizing behavior also did not predict subsequent fluctuations in parental knowledge (Hypothesis 3 not confirmed). Additionally, although children’s gender did not moderate the lagged associations, fluctuations in fathers’, but not mothers’, parental knowledge was negatively associated with subsequent fluctuations in children’s externalizing behavior (Hypothesis 4). Furthermore, exploratory analyses showed negative linear co-development of increasing parental knowledge and decreasing children’s externalizing behavior between families. Taken together, the results emphasize stable between-family differences, linear co-development, and the possible importance of fathers’ parental knowledge. In the following, these results are discussed in more detail.

### Stable between-Family Differences

As stated, the current study found stable negative between-family correlations between parental knowledge and children’s externalizing behavior. Such between-family correlations were also consistently found and researched in previous research (e.g., Micalizzi et al., 2019; Willoughby & Hamza, 2011). Likewise, the levels of parental knowledge and children’s externalizing behavior decreased generally over time, which is in line with the results of the meta-analysis of Lionetti et al. (2019). These negative correlations might reflect the influence of structural factors, such as socioeconomic status, genetics, and personality traits (Belsky & Pluess, 2009; Beyers et al., 2003). For example, adolescents’ stable levels of self-control could affect both parenting and delinquency (Denissen et al., 2009; Janssen et al., 2016), and overlapping genetic predispositions could evoke lower levels of parental knowledge and higher levels of children’s externalizing behavior (Kuo et al., 2021).

### Within-Family Cross-Lagged Effects

Mixed support for within-family lagged effects was found. When both mothers and fathers were investigated in the same model (see below for results on fathers), fluctuations in parental knowledge and children’s externalizing behavior did not predict each other when controlled for between-family differences and between-family linear co-development. Likewise, no moderation of age was found. This suggests an absence of within-family lagged effects between children’s externalizing behavior and parental knowledge, at least in the average family. The absence of average within-family lagged effects may be due to effect heterogeneity (Bolger et al., 2019). Because several within-family parenting studies show that families can differ in presence, strength, and sign of their parenting effects (i.e., positive and negative; Boele, Bülow, et al., 2022; Bülow et al., 2022), average effects might not be generalizable to every individual family (van Roekel et al., 2019) and may be less informative. Additionally, the studied annual timescale may be too large to capture parenting processes with parental knowledge (e.g., Boele, Nelemans, et al., 2022). Although theory is not particularly explicit about the relevant timescale of monitoring processes (Kerr et al., 2010; Patterson, 2009), effects between parental knowledge and children’s externalizing problem behavior might unfold over shorter timescales, such as days (e.g., Villalobos Solis et al., 2015), weeks, or months (e.g., Keijsers et al., 2016). Hence, future studies exploring and explaining the extent to which parenting processes with parental knowledge are heterogeneous between families and across timescales are necessary.

### Exploratory Findings

To describe the within-family developmental processes in full detail, this study also explored significantly correlated residuals and carry-over effects. First, significantly correlated residuals were likely found as more positive parenting behavior, including more parental knowledge, is generally related to lower levels of children’s externalizing behavior (Zhang et al., 2020). Second, residuals were somewhat stable over time: the carry-over effects showed that higher-than-typical scores of externalizing behavior consistently predicted subsequent higher-than-typical scores of externalizing behavior. For parental knowledge, stability of residuals was only found between ages 10–12. This suggests that, after controlling for linear development, fluctuations in parental knowledge were likely not retained across children’s development, whereas fluctuations in children’s externalizing behavior were retained somewhat longer, which is consistent with previous research (Serbin et al., 2015; Zhang et al., 2020). It might be that the level of parental knowledge is more fluid across adolescence, as children strive for more independence and search for balances in how much they tell their parents (Lionetti et al., 2019). Likewise, parents might search for balance in their monitoring as they are likely aware of their children’s strive for independence.

### Moderation by Children’s and Parents’ Gender

In addition, the potential moderator role of children’s and parents’ gender was explored. Although boys displayed, on average, more externalizing behavior and less carry-over effects, and parents of boys had less parental knowledge, no substantial moderation effects of children’s gender were found. Thus, inconsistent with the between-family study of Gryczkowski (2010) but consistent with the meta-analysis of Liu et al. (2020), no children’s gender differences were found for within-family lagged effects and between-family differences. Nevertheless, several parents’ gender differences were found. Within-family lagged effects were found for fathers as age-specific fluctuations in parent-specific mean levels of parental knowledge were negatively associated with subsequent age-specific fluctuations in child-specific mean levels of externalizing behavior for fathers, but not for mothers. This suggests that children’s externalizing behavior would decrease after fathers became more knowledgeable. Presumably, increases or decreases in mothers’ knowledge were not retained, whereas increases in fathers’ knowledge were retained somewhat longer and might have had a greater chance to predict subsequent levels of children’s externalizing behavior. These differences might emerge through lower involvement of fathers in children’s upbringing (Elam et al., 2017). As a result, fathers might have started with less knowledge, on average (Crouter et al., 1999). This might produce stable impressions of children’s behavior. Noteworthy is that the level of mothers’ knowledge was, on average, already high compared to fathers’ knowledge. Consequently, an increase in fathers’ knowledge might be more feasible as fathers might be able to improve substantially, whereas mothers might be able to improve less as they already have high levels of maternal knowledge.

Nevertheless, contrary to the results of Elam and colleagues (2017) and Zhang and colleagues (2020), no differences between mothers and fathers were found for child-driven increases in parental knowledge. The results of the current study might differ from these previous studies since this study examined a community sample displaying high mean levels of parental knowledge and low mean levels of externalizing behavior, whereas these previous authors examined high-risk samples displaying wider ranges of those mean levels and higher prevalence of problematic behavior. There might be more fluctuations within families and other parent-child dynamics might be influential. Therefore, the results might only be generalizable to normatively developing children, and parental behavior might affect adolescents differently in high-risk families.

### Implications for Developmental Theories

The lack of evidence for within-family bidirectional associations between parental knowledge and children’s externalizing behavior is inconsistent with previous theories (e.g., Kerr et al., 2010; Patterson, 2009), but is consistent with most research on within-family effects, which are often zero on average (Boele et al., 2020; Boele et al., 2022). One explanation might be that parental perceptions of their children are still influential – but perhaps more so in early childhood, when they are more in flux compared to later phases (Olson et al., 2000). Patterson himself proposed that vicious circles between reduced parental knowledge, reduced demands of adjustments of children’s behavior, and increased children’s externalizing behavior are resistant to extinction and are less likely to produce changes in behavioral development at later ages (Dishion & Snyder, 2016). Accordingly, stable development (i.e., reflected in associations between random intercepts and slopes) might be the result of long-time repetitions of coercive cycles at earlier ages. Additionally, it might also be that temporary deviations of an otherwise consistent pattern of parenting do not have large influences, because they are not consistently present by definition, and instead revert to stable development (Fuligni & Eccles, 1993). This is good news in the case of parental mistakes: Temporary deviations might not have a large impact when it occurs in a relatively good and stable parenting context.

### Strengths, Limitations, and Directions for Future Research

This study adds insights into the interplay of parental knowledge and children’s externalizing behavior within families. As a large representative sample of the German population was used, the results are potentially generalizable to other Western countries. The sample size and the number of waves were sufficient to implement RI-CLPMs to detect bidirectional effects at the within-family level and between-family linear co-development, something which previous studies often lacked (Marceau et al., 2015). Thereby, the exploration of effect heterogeneity in terms of age and sex differences of both parents and children are strengths of the current study.

However, this study also has some limitations. First, different aspects of parental knowledge should be measured to provide additional information. For example, additional inclusion of parental solicitation, control, and adolescent secrecy might provide insights into which variables provoke most developmental change in children’s externalizing behavior (Lionetti et al., 2019). Although multi-faceted measures likely provide more information about the different aspects of parental knowledge, the results likely provide a valid indication of global levels of parental knowledge (Florean et al., 2022). Second, the reliability coefficients of children’s externalizing behavior measurements were suboptimal, but similar to others that also included the subscale Conduct Problems (Essau et al., 2012) and concluded that the subscale is a valid instrument (Klasen et al., 2003). Nevertheless, results should be interpreted with caution as the examined associations might be attenuated. Third, incorporating multiple informants, like teachers or both parents, might be informative. There might be influential mismatches in parents’ and children’s perspectives regarding each other’s behavior, and parent-child interactions might be influenced by these different perspectives (Lionetti et al., 2019; Luan et al., 2017). Fourth, it might be that there are within-family effects at an individual level, but not at an average level as within-family effects might be heterogeneous (Keijsers et al., 2016). Fifth, as described above, the annual timescale might be too large to capture parenting processes. Last, future studies should try to replicate the studied associations during earlier and later ages. As discussed, stability patterns might reflect consolidated processes during earlier ages. Similarly, investigating the associations during late adolescence might again reveal notable differences. Increased parental knowledge might trigger reactance when aimed to decrease late adolescents’ or early adults’ externalizing behavior, as they strive for independence (Dishion & Snyder, 2016; Koepke & Denissen, 2012).

## Conclusion

Research has established that parental knowledge and children’s externalizing behavior are negatively associated at the between-family level. This study aimed to replicate these between-family differences, while also aiming to investigate within-family effects. The RI-CLPMs with random linear slopes indeed confirmed negative between-family associations but provided no evidence for bidirectional associations within families. That is, fluctuations in parental knowledge did not predict subsequent fluctuations in children’s externalizing behavior within families or vice versa. These results are inconsistent with theory, but consistent with recent within-family parenting research. Results did indicate linear co-development and carry-over effects within families of parental knowledge and children’s externalizing behavior. Moreover, although children’s gender did not moderate the lagged within-family effects, support was found for lagged effects of increased paternal knowledge, but not for maternal knowledge, predicting decreased children’s externalizing behavior one year later. The findings highlight the need to separately examine effects at the between- and within-family level, and of mother and fathers, when investigating parent-child bidirectionality.

## Supplementary Information

Supplementary material

## Footnotes

1 The constrained cross-lagged paths from parental knowledge to subsequent children’s externalizing behavior were significant in the multigroup model (*B* = −0.06, *SE* = 0.03, β = −0.04, *p* = 0.044). Nevertheless, we focused on the parameter estimates of the final RI-CLPM as the cross-lagged paths were invariant across gender.
