# Supplementary material for: Unraveling the Bidirectional Associations between Parental Knowledge and Children’s Externalizing Behavior
Source: J Youth Adolesc. 2023 Feb 15;52(4):794–809. doi: 10.1007/s10964-023-01743-4 (PMC9957859; doi:10.1007/s10964-023-01743-4)
Supplement: Supplementary file 1 — Supplementary material [file 10964_2023_1743_MOESM1_ESM.pdf]

Supplementary material

Supplementary table 1. Descriptive Statistics per Wave

Supplementary table 2. Between-Family Correlations for Parental Knowledge, Children's Externalizing Behavior, Children's and Parents' Gender

Supplementary text 1. Description of Moderation Results of Children's Gender and Parent's Gender

Supplementary table 3. Parameter Estimates of the Multi-Group RI-CLPM for Boys

Supplementary table 4. Parameter Estimates of the Multi-Group RI-CLPM for Girls

Supplementary table 5. Parameter Estimates of the Multi-Group RI-CLPM for Fathers

Supplementary table 6. Parameter Estimates of the Multi-Group RI-CLPM for Mothers

Supplementary text 2. Description of Sensitivity Analyses

Supplementary table 7. Model Summary Statistics and Model Comparisons of the Exploratory RI-CLPMs

Supplementary table 8. Parameter Estimates of the Exploratory RI-CLPM With Constrained Correlated Residuals and Cross-lagged Paths Across Age

Supplementary table 1. Descriptive Statistics per Wave

Variable	n_{total}	n_{boys}	n_{father}	M_{total}	SD_{total}
Parental knowledge					
Wave 2	689	361	183	4.20	0.58
Wave 3	860	449	241	4.21	0.56
Wave 4	953	486	287	4.19	0.56
Wave 5	1219	620	366	4.13	0.56
Wave 6	1420	719	456	4.07	0.58
Wave 7	1740	902	583	4.06	0.59
Wave 8	1706	877	560	4.07	0.61
Wave 9	1699	872	611	4.05	0.62
Wave 9	1482	755	527	4.02	0.64
Wave 10	1282	655	456	4.01	0.62
Externalizing behavior					
Wave 2	728	376	194	0.38	0.31
Wave 3	860	449	241	0.38	0.32
Wave 4	953	486	287	0.36	0.32
Wave 5	1218	619	365	0.35	0.31
Wave 6	1429	725	459	0.37	0.32
Wave 7	1750	907	586	0.36	0.32
Wave 8	1709	879	560	0.35	0.31
Wave 9	1699	872	611	0.33	0.31
Wave 9	1481	752	525	0.34	0.32
Wave 10	1278	652	452	0.31	0.31

Supplementary table 2. Between-Family Correlations for Parental Knowledge, Children's Externalizing Behavior, Children's and Parents' Gender

Variable	1	2	3	4	5	6	7	8	9	10	11	12	13	14	15	16	17
1. Kno. Age 8	—																
2. Kno. Age 9	.63*	—															
3. Kno. Age 10	.64*	.60*	—														
4. Kno. Age 11	.54*	.59*	.66*	—													
5. Kno. Age 12	.51*	.56*	.64*	.66*	—												
6. Kno. Age 13	.45*	.51*	.60*	.61*	.64*	—											
7. Kno. Age 14	.42*	.52*	.48*	.54*	.63*	.63*	—										
8. Kno. Age 15	.45*	.49*	.50*	.56*	.59*	.58*	.64*	—									
9. Ext. Age 8	-.19*	-.19*	-.19*	-.17*	-.18*	-.09*	-.07	-.23*	—								
10. Ext. Age 9	-.17*	-.23*	-.15*	-.17*	-.19*	-.18*	-.15*	-.19*	.63*	—							
11. Ext. Age 10	-.14*	-.17*	-.20*	-.17*	-.21*	-.17*	-.17*	-.17*	.62*	.61*	—						
12. Ext. Age 11	-.12*	-.18*	-.18*	-.20*	-.22*	-.20*	-.18*	-.25*	.58*	.57*	.66*	—					
13. Ext. Age 12	-.09*	-.14*	-.16*	-.13*	-.22*	-.23*	-.22*	-.16*	.53*	.53*	.60*	.62*	—				
14. Ext. Age 13	.07	-.06	-.06	-.11*	-.11*	-.19*	-.14*	-.14*	.39*	.50*	.48*	.56*	.62*	—			
15. Ext. Age 14	-.02	-.06	-.03	-.09*	-.16*	-.16*	-.21*	-.20*	.34*	.44*	.53*	.53*	.60*	.62*	—		
16. Ext. Age 15	-.18*	-.08	-.12*	-.09	-.09*	-.18*	-.23*	-.24*	.34*	.33*	.46*	.46*	.53*	.54*	.63*	—	
17. Parents' gender[a]	.37*	.33*	.30*	.28*	.30*	.23*	.27*	.20*	-.06*	-.04	-.02	-.00	-.02	.08*	-.01	-.02	—
18. Children's gender[b]	.05*	.11*	.09*	.05*	.09*	.07*	.08*	.10*	-.15*	-.14*	-.13*	-.12*	-.12*	-.06*	-.04	-.01	.03

Note. Estimates are Pearson correlations. Kno. = Parental knowledge. Ext. = Children's externalizing behavior. [a] Estimates are point-biserial correlations (0 = fathers, 1 = mothers). [b] Estimates are point-biserial correlations (0 = boys, 1 = girls).

*$p < .05$.

Supplementary text 1. Description of Moderation Results of Children's Gender and Parent's Gender

Moderating Role of Children's Gender

To explore children's gender as a moderator of the within-family associations (Hypothesis 4), we estimated several multi-group RI-CLPMs. The baseline model, the multigroup version (Model 5) of the final RI-CLPM (Model 4), indicated good fit to the data, see Table 2. Thereafter, the parameters were structurally constrained to be equal across gender to retain the most parsimonious model. Eventually, the baseline multigroup RI-CLPM with the intercept-intercept correlation, slope-slope correlation, and cross-lagged paths constrained across gender was investigated as most parsimonious model (Model 10). As shown in Table 2, Figure 3, and Supplementary Tables 3-4, statistically significant gender differences were only found between the correlated residuals (p = .019) and auto-regressive paths (p < .001) as these parameters were not constrained to be equal. The correlated residuals showed a smaller negative correlation for girls (β = -.08) than for boys (β = -.13), indicating that increased parental knowledge was related to a greater concurrent decrease in externalizing behavior for boys than girls. The one-year interval autoregressive paths of externalizing behavior between ages 12-13 and 13-14 were significant and positive for girls ($\beta_{12\text{-}13}$ = .27; $\beta_{13\text{-}14}$ = .29), but not for boys. Similarly, the autoregressive paths for parental knowledge between ages 8-9 and 13-14 were significant and positive for girls ($\beta_{8\text{-}9}$ = .12; $\beta_{13\text{-}14}$ = .14), but not for boys.

Moderating Role of Parents' Gender

To explore parents' gender as a moderator of the associations (Hypothesis 4), we estimated several multi-group RI-CLPMs. The baseline model, the multigroup version (Model 11) of the final RI-CLPM (Model 4), indicated good fit to the data, see Table 2. Thereafter, the parameters were structurally constrained to be equal across gender. The baseline multigroup RI-CLPM with the intercept-intercept and slope-slope correlation constrained across gender (Model 13) was investigated as the most parsimonious model. As shown in Table 2, Figure 4, and Supplementary Tables 5-6, statistically significant gender differences were only found between the correlated residuals (p < .001), autoregressive paths (p < .001), and cross-lagged paths (p = .012) as these parameters were not constrained to be equal. The correlated residuals showed a smaller negative correlation for mothers (β = -.09) than for fathers (β = -.11), indicating that greater decreases in externalizing behavior were present when fathers, compared to mothers, increased their knowledge. The one-year interval autoregressive paths of children's externalizing behavior between ages 11-14 were significant and positive for fathers ($\beta_{11\text{-}12}$ = .13; $\beta_{12\text{-}13}$ = .21; $\beta_{13\text{-}14}$ = .27) but not for mothers, and the path between ages 14-15 was significant and positive

for mothers (β = .40) but not for fathers. Similarly, the path for parental knowledge between ages 11-12 was significant and positive for fathers (β = .14), but not for mothers. The path between ages 14-15 was significant for both parents combined but masked a negative coefficient for fathers (β = -.18) and a positive one for mothers (β = .30).

Supplementary table 3. Parameter Estimates of the Multi-Group RI-CLPM for Boys

Parameter	*B*	*SE B*	β	*z*	*p*	95% CI for *B*
Autoregressive paths						
Ext. Age 8 → Ext. Age 9	0.26	0.06	.26	4.58	<.001	[0.15, 0.37]
Ext. Age 9 → Ext. Age 10	0.16	0.05	.16	3.21	.001	[0.06, 0.25]
Ext. Age 10 → Ext. Age 11	0.25	0.05	.25	5.07	<.001	[0.16, 0.35]
Ext. Age 11 → Ext. Age 12	0.14	0.06	.14	2.33	.020	[0.02, 0.26]
Ext. Age 12 → Ext. Age 13	0.08	0.06	.08	1.39	.165	[-0.03, 0.20]
Ext. Age 13 → Ext. Age 14	0.06	0.07	.06	0.88	.378	[-0.07, 0.19]
Ext. Age 14 → Ext. Age 15	0.26	0.08	.25	3.44	.001	[0.11, 0.41]
Kno. Age 8 → Kno. Age 9	-0.03	0.07	-.03	-0.48	.630	[-0.17, 0.10]
Kno. Age 9 → Kno. Age 10	-0.07	0.05	-.07	-1.4	.163	[-0.18, 0.03]
Kno. Age 10 → Kno. Age 11	0.12	0.05	.12	2.39	.017	[0.02, 0.22]
Kno. Age 11 → Kno. Age 12	0.13	0.05	.13	2.49	.013	[0.03, 0.24]
Kno. Age 12 → Kno. Age 13	0.06	0.06	.06	1.03	.302	[-0.06, 0.18]
Kno. Age 13 → Kno. Age 14	0.01	0.06	.01	0.16	.873	[-0.12, 0.14]
Kno. Age 14 → Kno. Age 15	-0.06	0.08	-.06	-0.72	.471	[-0.21, 0.10]
Cross-lagged paths						
Ext. Age 8 → Kno. Age 9	0.00[a]	0.01	.01	0.33	.740	[-0.02, 0.02]
Ext. Age 9 → Kno. Age 10	0.00[a]	0.01	.01	0.33	.740	[-0.02, 0.02]
Ext. Age 10 → Kno. Age 11	0.00[a]	0.01	.01	0.33	.740	[-0.02, 0.02]
Ext. Age 11 → Kno. Age 12	0.00[a]	0.01	.01	0.33	.740	[-0.02, 0.02]
Ext. Age 12 → Kno. Age 13	0.00[a]	0.01	.01	0.33	.740	[-0.02, 0.02]
Ext. Age 13 → Kno. Age 14	0.00[a]	0.01	.01	0.33	.740	[-0.02, 0.02]
Ext. Age 14 → Kno. Age 15	0.00[a]	0.01	.01	0.33	.740	[-0.02, 0.02]
Kno. Age 8 → Ext. Age 9	-0.06[b]	0.03	-.04	-2.02	.044	[-0.12, -0.00]
Kno. Age 9 → Ext. Age 10	-0.06[b]	0.03	-.04	-2.02	.044	[-0.12, -0.00]
Kno. Age 10 → Ext. Age 11	-0.06[b]	0.03	-.04	-2.02	.044	[-0.12, -0.00]
Kno. Age 11 → Ext. Age 12	-0.06[b]	0.03	-.04	-2.02	.044	[-0.12, -0.00]
Kno. Age 12 → Ext. Age 13	-0.06[b]	0.03	-.04	-2.02	.044	[-0.12, -0.00]
Kno. Age 13 → Ext. Age 14	-0.06[b]	0.03	-.04	-2.02	.044	[-0.12, -0.00]
Kno. Age 14 → Ext. Age 15	-0.06[b]	0.03	-.04	-2.02	.044	[-0.12, -0.00]
Correlations						
Correlation Age 8	-0.01	0.00	-.17	-3.23	.001	[-0.02, -0.01]
Residual correlation Age 9	-0.01	0.00	-.13	-6.40	<.001	[-0.01, -0.01]
Residual correlation Age 10	-0.01	0.00	-.13	-6.40	<.001	[-0.01, -0.01]
Residual correlation Age 11	-0.01	0.00	-.13	-6.40	<.001	[-0.01, -0.01]
Residual correlation Age 12	-0.01	0.00	-.13	-6.40	<.001	[-0.01, -0.01]
Residual correlation Age 13	-0.01	0.00	-.13	-6.40	<.001	[-0.01, -0.01]
Residual correlation Age 14	-0.01	0.00	-.13	-6.40	<.001	[-0.01, -0.01]
Residual correlation Age 15	-0.01	0.00	-.13	-6.40	<.001	[-0.01, -0.01]
Intercept-intercept correlation	-0.02[c]	0.00	-.22	-9.12	<.001	[-0.03, -0.02]
Slope-Slope correlation	-0.00[d]	0.00	-.64	-4.93	<.001	[-0.00, 0.00]

Note. Kno. = Parental knowledge. Ext. = Children’s externalizing behavior. CI = Confidence interval. Superscripts indicate that parameters have been set equal across children’s gender.

Supplementary table 4. Parameter Estimates of the Multi-Group RI-CLPM for Girls

Parameter	*B*	*SE B*	β	*z*	*p*	95% CI for *B*
Autoregressive paths						
Ext. Age 8 → Ext. Age 9	0.18	0.06	.18	3.15	.002	[0.07, 0.29]
Ext. Age 9 → Ext. Age 10	0.13	0.05	.13	2.46	.014	[0.03, 0.23]
Ext. Age 10 → Ext. Age 11	0.20	0.06	.20	3.63	<.001	[0.09, 0.31]
Ext. Age 11 → Ext. Age 12	0.11	0.05	.11	2.24	.025	[0.01, 0.21]
Ext. Age 12 → Ext. Age 13	0.28	0.07	.27	4.20	<.001	[0.15, 0.41]
Ext. Age 13 → Ext. Age 14	0.29	0.06	.29	4.54	<.001	[0.17, 0.42]
Ext. Age 14 → Ext. Age 15	0.19	0.08	.19	2.28	.023	[0.03, 0.35]
Kno. Age 8 → Kno. Age 9	0.13	0.06	.12	1.99	.047	[0.00, 0.26]
Kno. Age 9 → Kno. Age 10	0.04	0.06	.04	0.73	.465	[-0.07, 0.15]
Kno. Age 10 → Kno. Age 11	0.21	0.06	.21	3.88	<.001	[0.11, 0.32]
Kno. Age 11 → Kno. Age 12	0.13	0.05	.13	2.47	.013	[0.03, 0.23]
Kno. Age 12 → Kno. Age 13	0.07	0.06	.07	1.08	.279	[-0.05, 0.19]
Kno. Age 13 → Kno. Age 14	0.14	0.07	.14	2.05	.041	[0.01, 0.27]
Kno. Age 14 → Kno. Age 15	0.01	0.08	.01	0.16	.876	[-0.14, 0.16]
Cross-lagged paths						
Ext. Age 8 → Kno. Age 9	0.00[a]	0.01	.01	0.33	.740	[-0.02, 0.02]
Ext. Age 9 → Kno. Age 10	0.00[a]	0.01	.01	0.33	.740	[-0.02, 0.02]
Ext. Age 10 → Kno. Age 11	0.00[a]	0.01	.01	0.33	.740	[-0.02, 0.02]
Ext. Age 11 → Kno. Age 12	0.00[a]	0.01	.01	0.33	.740	[-0.02, 0.02]
Ext. Age 12 → Kno. Age 13	0.00[a]	0.01	.01	0.33	.740	[-0.02, 0.02]
Ext. Age 13 → Kno. Age 14	0.00[a]	0.01	.01	0.33	.740	[-0.02, 0.02]
Ext. Age 14 → Kno. Age 15	0.00[a]	0.01	.01	0.33	.740	[-0.02, 0.02]
Kno. Age 8 → Ext. Age 9	-0.06[b]	0.03	-.04	-2.02	.044	[-0.12, -0.00]
Kno. Age 9 → Ext. Age 10	-0.06[b]	0.03	-.03	-2.02	.044	[-0.12, -0.00]
Kno. Age 10 → Ext. Age 11	-0.06[b]	0.03	-.03	-2.02	.044	[-0.12, -0.00]
Kno. Age 11 → Ext. Age 12	-0.06[b]	0.03	-.03	-2.02	.044	[-0.12, -0.00]
Kno. Age 12 → Ext. Age 13	-0.06[b]	0.03	-.03	-2.02	.044	[-0.12, -0.00]
Kno. Age 13 → Ext. Age 14	-0.06[b]	0.03	-.04	-2.02	.044	[-0.12, -0.00]
Kno. Age 14 → Ext. Age 15	-0.06[b]	0.03	-.04	-2.02	.044	[-0.12, -0.00]
Correlations						
Correlation Age 8	0.00	0.00	-.03	-0.50	.618	[-0.01, 0.01]
Residual correlation Age 9	-0.01	0.00	-.08	-4.23	<.001	[-0.01, -0.00]
Residual correlation Age 10	-0.01	0.00	-.08	-4.23	<.001	[-0.01, -0.00]
Residual correlation Age 11	-0.01	0.00	-.08	-4.23	<.001	[-0.01, -0.00]
Residual correlation Age 12	-0.01	0.00	-.08	-4.23	<.001	[-0.01, -0.00]
Residual correlation Age 13	-0.01	0.00	-.08	-4.23	<.001	[-0.01, -0.00]
Residual correlation Age 14	-0.01	0.00	-.08	-4.23	<.001	[-0.01, -0.00]
Residual correlation Age 15	-0.01	0.00	-.08	-4.23	<.001	[-0.01, -0.00]
Intercept-intercept correlation	-0.02[c]	0.00	-.27	-9.12	<.001	[-0.03, -0.02]
Slope-Slope correlation	-0.00[d]	0.00	-.54	-4.93	<.001	[-0.00, 0.00]

Note. Kno. = Parental knowledge. Ext. = Children's externalizing behavior. CI = Confidence interval. Superscripts indicate that parameters have been set equal across children's gender.

Supplementary table 5. Parameter Estimates of the Multi-Group RI-CLPM for Fathers

Parameter	*B*	*SE B*	β	*z*	*p*	95% CI for *B*
Autoregressive paths						
Ext. Age 8 → Ext. Age 9	0.19	0.05	.20	3.84	<.001	[0.09, 0.29]
Ext. Age 9 → Ext. Age 10	0.13	0.04	.13	2.92	.004	[0.04, 0.21]
Ext. Age 10 → Ext. Age 11	0.24	0.04	.24	5.47	<.001	[0.16, 0.33]
Ext. Age 11 → Ext. Age 12	0.13	0.05	.13	2.88	.004	[0.04, 0.22]
Ext. Age 12 → Ext. Age 13	0.21	0.06	.21	3.76	<.001	[0.10, 0.32]
Ext. Age 13 → Ext. Age 14	0.28	0.05	.27	5.14	<.001	[0.17, 0.38]
Ext. Age 14 → Ext. Age 15	0.12	0.07	.12	1.65	.099	[-0.02, 0.25]
Kno. Age 8 → Kno. Age 9	0.00	0.06	.00	-0.03	.978	[-0.12, 0.12]
Kno. Age 9 → Kno. Age 10	-0.02	0.04	-.02	-0.5	.619	[-0.11, 0.06]
Kno. Age 10 → Kno. Age 11	0.13	0.04	.13	2.93	.003	[0.04, 0.22]
Kno. Age 11 → Kno. Age 12	0.12	0.04	.12	2.8	.005	[0.04, 0.21]
Kno. Age 12 → Kno. Age 13	0.07	0.05	.07	1.32	.186	[-0.03, 0.17]
Kno. Age 13 → Kno. Age 14	0.06	0.05	.06	1.09	.274	[-0.05, 0.17]
Kno. Age 14 → Kno. Age 15	-0.19	0.07	-.18	-2.78	.006	[-0.32, -0.06]
Cross-lagged paths						
Ext. Age 8 → Kno. Age 9	-0.01	0.01	-.02	-0.92	.360	[-0.04, 0.01]
Ext. Age 9 → Kno. Age 10	-0.01	0.01	-.02	-0.92	.360	[-0.04, 0.01]
Ext. Age 10 → Kno. Age 11	-0.01	0.01	-.02	-0.92	.360	[-0.04, 0.01]
Ext. Age 11 → Kno. Age 12	-0.01	0.01	-.02	-0.92	.360	[-0.04, 0.01]
Ext. Age 12 → Kno. Age 13	-0.01	0.01	-.02	-0.92	.360	[-0.04, 0.01]
Ext. Age 13 → Kno. Age 14	-0.01	0.01	-.02	-0.92	.360	[-0.04, 0.01]
Ext. Age 14 → Kno. Age 15	-0.01	0.01	-.02	-0.92	.360	[-0.04, 0.01]
Kno. Age 8 → Ext. Age 9	-0.11	0.03	-.07	-3.28	.001	[-0.18, -0.05]
Kno. Age 9 → Ext. Age 10	-0.11	0.03	-.07	-3.28	.001	[-0.18, -0.05]
Kno. Age 10 → Ext. Age 11	-0.11	0.03	-.07	-3.28	.001	[-0.18, -0.05]
Kno. Age 11 → Ext. Age 12	-0.11	0.03	-.07	-3.28	.001	[-0.18, -0.05]
Kno. Age 12 → Ext. Age 13	-0.11	0.03	-.07	-3.28	.001	[-0.18, -0.05]
Kno. Age 13 → Ext. Age 14	-0.11	0.03	-.07	-3.28	.001	[-0.18, -0.05]
Kno. Age 14 → Ext. Age 15	-0.11	0.03	-.07	-3.28	.001	[-0.18, -0.05]
Correlations						
Correlation Age 8	-0.01	0.00	-.14	-2.86	.004	[-0.02, -0.00]
Residual correlation Age 9	-0.01	0.00	-.11	-6.32	<.001	[-0.01, -0.01]
Residual correlation Age 10	-0.01	0.00	-.11	-6.32	<.001	[-0.01, -0.01]
Residual correlation Age 11	-0.01	0.00	-.11	-6.32	<.001	[-0.01, -0.01]
Residual correlation Age 12	-0.01	0.00	-.11	-6.32	<.001	[-0.01, -0.01]
Residual correlation Age 13	-0.01	0.00	-.11	-6.32	<.001	[-0.01, -0.01]
Residual correlation Age 14	-0.01	0.00	-.11	-6.32	<.001	[-0.01, -0.01]
Residual correlation Age 15	-0.01	0.00	-.11	-6.32	<.001	[-0.01, -0.01]
Intercept-intercept correlation	-0.02[a]	0.00	-.27	-9.39	<.001	[-0.03, -0.02]
Slope-Slope correlation	-0.00[b]	0.00	-.52	-5.24	<.001	[-0.00, 0.00]

Note. Kno. = Parental knowledge. Ext. = Children's externalizing behavior. CI = Confidence interval. Superscripts indicate that parameters have been set equal across parents' gender.

Supplementary table 6. Parameter Estimates of the Multi-Group RI-CLPM for Mothers

Parameter	*B*	*SE B*	β	*z*	*p*	95% CI for *B*
Autoregressive paths						
Ext. Age 8 → Ext. Age 9	0.27	0.07	.27	4.04	<.001	[0.14, 0.41]
Ext. Age 9 → Ext. Age 10	0.19	0.06	.19	3.07	.002	[0.07, 0.31]
Ext. Age 10 → Ext. Age 11	0.21	0.07	.21	3.17	.002	[0.08, 0.34]
Ext. Age 11 → Ext. Age 12	0.11	0.07	.11	1.48	.138	[-0.03, 0.25]
Ext. Age 12 → Ext. Age 13	0.06	0.07	.06	0.86	.392	[-0.08, 0.21]
Ext. Age 13 → Ext. Age 14	-0.07	0.09	-.07	-0.75	.455	[-0.24, 0.11]
Ext. Age 14 → Ext. Age 15	0.43	0.09	.40	4.93	<.001	[0.26, 0.60]
Kno. Age 8 → Kno. Age 9	0.09	0.08	.08	1.08	.279	[-0.07, 0.24]
Kno. Age 9 → Kno. Age 10	0.03	0.07	.03	0.43	.664	[-0.11, 0.17]
Kno. Age 10 → Kno. Age 11	0.24	0.07	.23	3.48	<.001	[0.10, 0.37]
Kno. Age 11 → Kno. Age 12	0.14	0.07	.14	1.9	.057	[-0.00, 0.27]
Kno. Age 12 → Kno. Age 13	0.06	0.08	.06	0.69	.492	[-0.10, 0.22]
Kno. Age 13 → Kno. Age 14	0.09	0.09	.09	1.03	.302	[-0.08, 0.26]
Kno. Age 14 → Kno. Age 15	0.31	0.10	.30	3.26	.001	[0.12, 0.50]
Cross-lagged paths						
Ext. Age 8 → Kno. Age 9	0.03	0.02	.05	1.84	.065	[-0.00, 0.06]
Ext. Age 9 → Kno. Age 10	0.03	0.02	.05	1.84	.065	[-0.00, 0.06]
Ext. Age 10 → Kno. Age 11	0.03	0.02	.05	1.84	.065	[-0.00, 0.06]
Ext. Age 11 → Kno. Age 12	0.03	0.02	.06	1.84	.065	[-0.00, 0.06]
Ext. Age 12 → Kno. Age 13	0.03	0.02	.06	1.84	.065	[-0.00, 0.06]
Ext. Age 13 → Kno. Age 14	0.03	0.02	.06	1.84	.065	[-0.00, 0.06]
Ext. Age 14 → Kno. Age 15	0.03	0.02	.05	1.84	.065	[-0.00, 0.06]
Kno. Age 8 → Ext. Age 9	0.05	0.05	.03	0.93	.352	[-0.06, 0.16]
Kno. Age 9 → Ext. Age 10	0.05	0.05	.03	0.93	.352	[-0.06, 0.16]
Kno. Age 10 → Ext. Age 11	0.05	0.05	.03	0.93	.352	[-0.06, 0.16]
Kno. Age 11 → Ext. Age 12	0.05	0.05	.03	0.93	.352	[-0.06, 0.16]
Kno. Age 12 → Ext. Age 13	0.05	0.05	.03	0.93	.352	[-0.06, 0.16]
Kno. Age 13 → Ext. Age 14	0.05	0.05	.03	0.93	.352	[-0.06, 0.16]
Kno. Age 14 → Ext. Age 15	0.05	0.05	.03	0.93	.352	[-0.06, 0.16]
Correlations						
Correlation Age 8	0.00	0.01	-.01	-0.17	.864	[-0.01, 0.01]
Residual correlation Age 9	-0.01	0.00	-.09	-3.53	<.001	[-0.01, -0.00]
Residual correlation Age 10	-0.01	0.00	-.09	-3.53	<.001	[-0.01, -0.00]
Residual correlation Age 11	-0.01	0.00	-.09	-3.53	<.001	[-0.01, -0.00]
Residual correlation Age 12	-0.01	0.00	-.09	-3.53	<.001	[-0.01, -0.00]
Residual correlation Age 13	-0.01	0.00	-.09	-3.53	<.001	[-0.01, -0.00]
Residual correlation Age 14	-0.01	0.00	-.09	-3.53	<.001	[-0.01, -0.00]
Residual correlation Age 15	-0.01	0.00	-.09	-3.53	<.001	[-0.01, -0.00]
Intercept-intercept correlation	-0.02[a]	0.00	-.23	-9.39	<.001	[-0.03, -0.02]
Slope-Slope correlation	-0.00[b]	0.00	-1.26	-5.24	<.001	[-0.00, 0.00]

Note. Kno. = Parental knowledge. Ext. = Children's externalizing behavior. CI = Confidence interval. Superscripts indicate that parameters have been set equal across parents' gender.

Supplementary text 2. Description of Sensitivity Analyses

Extra exploratory models, RI-CLPMs of the main model with incorporation of the slope-slope, intercept-intercept, and intercept-slope correlations were run. Multiple models were estimated with constrained parameters across age in the following order: constrained correlated residuals, autoregressive paths, and cross-lagged paths. These models were compared to retain the most parsimonious model with the RI-CLPM without constraints as baseline model. Model comparisons showed similar results to the comparisons of the RI-CLPMs of the main study, see Supplementary Table 7. Model estimates of the final model showed that the autoregressive paths from children's externalizing behavior between ages 8 & 9, and 9 & 10 and the correlation at Age 8 became insignificant, see Supplementary Table 8. The intercept-slope paths for children's externalizing behavior and parental knowledge were both significant and negative, indicating that higher initial levels were generally related to smaller linear decreases over time. The association between the intercept of children's externalizing behavior and slope of parental knowledge was insignificant, indicating that initial levels of children's externalizing behavior were generally not related to linear decreases in parental knowledge. The association between the intercept of parental knowledge and slope of children's externalizing behavior was significant and positive indicating that higher initial levels of parental knowledge were related to larger linear decreases in children's externalizing behavior in general.

Supplementary table 7. Model Summary Statistics and Model Comparisons of the Exploratory RI-CLPMs

Model	χ^2	*df*	CFI	TLI	RMSEA	SRMR	Model comparison	$\Delta\chi^2$	Δdf	*p*
1. RI-CLPM	122.70	74	0.99	0.99	0.01	0.03				
2. Con. correlated residuals	174.83	104	0.99	0.99	0.01	0.03	1 vs. 2	19.46	18	.364
3. Con. autoregressive paths	157.48	104	0.99	0.99	0.01	0.03	2 vs. 3	32.66	12	.001
4. Con. cross-lagged paths	142.17	92	0.99	0.99	0.01	0.03	2 vs. 4	15.31	12	.225

Note. RI-CLPM = Random-Intercept Cross-Lagged Panel Model. Con. = Constrained. CFI = Comparative fit index. TLI = Tucker-Lewis index. RMSEA = Root-mean-square error of approximation. SRMR = Standardized root-mean-square residual. CFI, TLI, RMSEA, and SRMR are model fit measures.

All χ^2-statistics were significant, $p < .001$.

Supplementary table 8. Parameter Estimates of the Exploratory RI-CLPM With Constrained Correlated Residuals and Cross-lagged Paths Across Age

Parameter	*B*	*SE B*	β	*z*	*p*	95% CI for *B*
Autoregressive paths						
Ext. Age 8 → Ext. Age 9	0.03	0.06	0.02	0.45	.655	[-0.09, 0.14]
Ext. Age 9 → Ext. Age 10	0.06	0.04	0.06	1.65	.099	[-0.01, 0.14]
Ext. Age 10 → Ext. Age 11	0.21	0.04	0.21	5.66	<.001	[0.14, 0.29]
Ext. Age 11 → Ext. Age 12	0.11	0.04	0.11	2.88	.004	[0.04, 0.19]
Ext. Age 12 → Ext. Age 13	0.14	0.04	0.14	3.10	.002	[0.05, 0.22]
Ext. Age 13 → Ext. Age 14	0.11	0.05	0.11	2.42	.015	[0.02, 0.20]
Ext. Age 14 → Ext. Age 15	0.14	0.06	0.14	2.36	.018	[0.02, 0.25]
Kno. Age 8 → Kno. Age 9	0.00	0.06	0.00	-0.03	.977	[-0.11, 0.11]
Kno. Age 9 → Kno. Age 10	-0.04	0.04	-0.04	-0.91	.361	[-0.11, 0.04]
Kno. Age 10 → Kno. Age 11	0.16	0.04	0.16	4.25	<.001	[0.09, 0.24]
Kno. Age 11 → Kno. Age 12	0.12	0.04	0.12	3.28	.001	[0.05, 0.20]
Kno. Age 12 → Kno. Age 13	0.05	0.04	0.05	1.23	.219	[-0.03, 0.14]
Kno. Age 13 → Kno. Age 14	0.06	0.05	0.06	1.28	.201	[-0.03, 0.15]
Kno. Age 14 → Kno. Age 15	-0.04	0.06	-0.04	-0.80	.423	[-0.15, 0.06]
Cross-lagged paths						
Ext. Age 8 → Kno. Age 9	-0.01[a]	0.03	-0.01	-0.41	.678	[-0.08, 0.05]
Ext. Age 9 → Kno. Age 10	-0.01[a]	0.03	-0.01	-0.41	.678	[-0.08, 0.05]
Ext. Age 10 → Kno. Age 11	-0.01[a]	0.03	-0.01	-0.41	.678	[-0.08, 0.05]
Ext. Age 11 → Kno. Age 12	-0.01[a]	0.03	-0.01	-0.41	.678	[-0.08, 0.05]
Ext. Age 12 → Kno. Age 13	-0.01[a]	0.03	-0.01	-0.41	.678	[-0.08, 0.05]
Ext. Age 13 → Kno. Age 14	-0.01[a]	0.03	-0.01	-0.41	.678	[-0.08, 0.05]
Ext. Age 14 → Kno. Age 15	-0.01[a]	0.03	-0.01	-0.41	.678	[-0.08, 0.05]
Kno. Age 8 → Ext. Age 9	0.01[b]	0.01	0.01	0.74	.458	[-0.01, 0.03]
Kno. Age 9 → Ext. Age 10	0.01[b]	0.01	0.01	0.74	.458	[-0.01, 0.03]
Kno. Age 10 → Ext. Age 11	0.01[b]	0.01	0.01	0.74	.458	[-0.01, 0.03]
Kno. Age 11 → Ext. Age 12	0.01[b]	0.01	0.01	0.74	.458	[-0.01, 0.03]
Kno. Age 12 → Ext. Age 13	0.01[b]	0.01	0.01	0.74	.458	[-0.01, 0.03]
Kno. Age 13 → Ext. Age 14	0.01[b]	0.01	0.01	0.74	.458	[-0.01, 0.03]
Kno. Age 14 → Ext. Age 15	0.01[b]	0.01	0.01	0.74	.458	[-0.01, 0.03]
Correlations						
Correlation Age 8	0.00	0.00	-0.04	-0.81	.418	[-0.01, 0.00]
Residual correlation Age 9	-0.01[c]	0.00	-0.09	-5.72	<.001	[-0.01, 0.00]
Residual correlation Age 10	-0.01[c]	0.00	-0.09	-5.72	<.001	[-0.01, 0.00]
Residual correlation Age 11	-0.01[c]	0.00	-0.09	-5.72	<.001	[-0.01, 0.00]
Residual correlation Age 12	-0.01[c]	0.00	-0.09	-5.72	<.001	[-0.01, 0.00]
Residual correlation Age 13	-0.01[c]	0.00	-0.09	-5.72	<.001	[-0.01, 0.00]
Residual correlation Age 14	-0.01[c]	0.00	-0.09	-5.72	<.001	[-0.01, 0.00]
Residual correlation Age 15	-0.01[c]	0.00	-0.09	-5.72	<.001	[-0.01, 0.00]
Intercept-intercept correlation	-0.03	0.00	-0.29	-9.20	<.001	[-0.04, -003]
Slope-Slope correlation	0.00	0.00	-0.49	-5.44	<.001	[0.00, 0.00]
Intercept Kno. - Slope Kno. correlation	0.00	0.00	-0.16	-2.49	.013	[-0.01, 0.00]
Intercept Ext. - Slope Ext. correlation	0.00	0.00	-0.43	-6.01	<.001	[0.00, 0.00]
Intercept Ext. - Slope Kno. correlation	0.00	0.00	0.00	-0.03	.975	[0.00, 0.00]
Intercept Kno. – Slope Ext. correlation	0.00	0.00	0.32	5.46	<.001	[0.00, 0.01]

Note. Kno. = Parental knowledge. Ext. = Children's externalizing behavior. CI = Confidence interval. Superscripts indicate that parameters could be constrained as equal across age.
